# The Storm on Hospital Sunday

**Published:** 1912-06-29

**Authors:** Edmund Hay Currie

**Affiliations:** Mansion House


### THE EDITOR'S LETTER-BOX.

### The Storm on Hospital Sunday.

*To the Editor of* The Hospital.

Sir,—With reference to the accompanying list of collections, the fierce storm on Hospital Sunday morning had a disastrous effect. The morning collection at St. Paul's Cathedral suffered considerably, but from another cause.

Will your readers assist to make up the large deficiency by forwarding help to the clergyman or minister of their place of worship, or to the Lord Mayor at the Mansion House, E.C.?

It is sad, indeed, if our hospitals are to suffer so much by the caprice of our climate. *Bis dat qui cito dat.*—Yours very faithfully, Edmund Hay Currie.

Mansion House, June 21.

[The list referred to appears on page 338.—Ed. The Hospital.]

